# Dexmedetomidine reduces lipopolysaccharide induced neuroinflammation, sickness behavior, and anhedonia

**DOI:** 10.1371/journal.pone.0191070

**Published:** 2018-01-19

**Authors:** Ching-Hua Yeh, Liang-Po Hsieh, Ming-Chung Lin, Tsui-Shan Wei, Hui-Ching Lin, Chia-Cheng Chang, Chung-Hsi Hsing

**Affiliations:** 1 Department of Medicinal Botanicals and Health applications, Da-Yeh University, Changhua, Taiwan; 2 Department of Medical Research, China Medical University Hospital, China Medical University, Taichung, Taiwan; 3 Department of Medical Research, Chi-Mei Medical Center, Tainan, Taiwan; 4 Department of Neurology, Cheng Ching General Hospital, Taichung, Taiwan; 5 Department of Anesthesiology, Chi-Mei Medical Center, Tainan, Taiwan; 6 Department and Institute of Physiology, School of Medicine, and Brain Research Center, National Yang-Ming University, Taipei, Taiwan; 7 Neural Regenerative Medicine/Center for Neurotrauma and Neuroregeneration, College of Medical Science and Technology, Taipei Medical University, Taipei, Taiwan; Massachusetts General Hospital, UNITED STATES

## Abstract

**Background:**

Peripheral innate immune response may induce sickness behavior through activating microglia, excessive cytokines production, and neuroinflammation. Dexmedetomidine (Dex) has anti-inflammatory effect. We investigated the effects of Dex on lipopolysaccharide (LPS)-induced neuroinflammation and sickness behavior in mice.

**Materials and methods:**

BALB/c mice were intraperitoneally (i.p.) injected with Dex (50 ug/kg) or vehicle. One hour later, the mice were injected (i.p.) with *Escherichia coli* LPS (0.33 mg/kg) or saline (n = 6 in each group). We analyzed the food and water intake, body weight loss, and sucrose preference of the mice for 24h. We also determined microglia activation and cytokines expression in the brains of the mice. In vitro, we determine cytokines expression in LPS-treated BV-2 microglial cells with or without Dex treatment.

**Results:**

In the Dex-pretreated mice, LPS-induced sickness behavior (anorexia, weight loss, and social withdrawal) were attenuated and microglial activation was lower than vehicle control. The mRNA expression of TNF-α, MCP-1, indoleamine 2, 3 dioxygenase (IDO), caspase-3, and iNOS were increased in the brain of LPS-challenged mice, which were reduced by Dex but not vehicle.

**Conclusion:**

Dexmedetomidine diminished LPS-induced neuroinflammation in the mouse brain and modulated the cytokine-associated changes in sickness behavior.

## Introduction

The symptoms of sickness, such as the loss of appetite, decline in cognitive function, sleepiness, withdrawal from normal social activities, fever, aching joints and fatigue was defined as sickness behavior. The sickness behavior occurs during infections by pathogenic microorganisms [1–3]. Sepsis is a systemic inflammatory response to infection and septic patients have high risk of developing multiple organ failure. Clinical studies have shown that up to 70% of septic patients develop cognitive deficits and memory loss [4, 5]. The mechanisms of sickness behavior by pathogen infection remain to be elucidated. The response for immune-induced immunological, physiological, and behavioral changes is necessary to communication between the immune system and the central nervous system (CNS). Activation of the peripheral innate immune system stimulates cytokine secretion in the CNS [6]. Neuroinflammation is a multiple immune response against the harmful effects of different stimulation within the CNS. Several studies suggest the pro-inflammatory cytokines tumor necrosis factor (TNF)-α, indoleamine 2,3-dioxygenase (IDO1), and monocyte chemotactic protein (MCP)-1 involved in sickness behavior [7–11]. The overexpression of inflammatory cytokines in the brain is associated with cognitive dysfunction, sickness behavior, and depression [12–14].

Previous studies suggest that microglial cells play a role in mediating behavioral changes in systemic infections [15–17]. Microglia cells are sentinels for lipopolysaccharide (LPS)-mediated inflammation and involved in both innate and adaptive immune responses in the CNS [15–17]. Iba1 (ionized calcium-binding adapter molecule 1) protein is highly and specifically expressed in monocytic cell lines and cultured microglia and may be used as a marker for detecting the activation of microglia by Immunohistochemistry [18]. Iba1 is involved in the Rho family of small GTPase, Rac, and calcium signaling pathways and was strongly upregulated in activated microglia which may be required for cell mobility and phagocytosis [19]. Microglia cells produce excessive amount of pro-inflammatory cytokines such as TNF-α, interleukin (IL)-1β, and nitric oxide (NO) as well as reactive oxygen species that can inhibit neurogenesis and induce neural cells apoptosis [15–17, 20, 21]. The production of oxidative and neuroactive mediators that may influence behavior were contributed in active microglia. These produced mediators not only regulate further immune response but also affect CNS function. This effect on the CNS function has been posited to cause sickness behavior [22–24]. For instance, IDO1 mediate tryptophan degradation and involved in behavior complication concomitant with inflammation [10]. Microglia-derived TNF-α can regulate neuronal cell death via caspase and Bcl2 family mediated apoptosis [25]. NO is the products of inducible nitric oxide synthase (iNOS) and increase in activated microglia cells in neuroinflammation [20]. Because activated microglia and excessive inflammatory cytokines were supposed to cause or exacerbate sickness behavior, it is a strategy to suppress microglia cells activity and production of oxidative and inflammatory mediators.

Dexmedetomidine (Dex), an α2-adrenoceptor (α2-AR) agonist, is used as an analgesic and sedative agent in the intensive care unit. Dex activated α2 receptors and inhibited neuronal firing, causing hypotension, bradycardia, sedation, and analgesia in the CNS and spinal cord [26, 27]. Also, Dex have been used as a preventative measure of and treatment modality for emergence delirium after general anesthesia [28]. Dex produced its neuroprotective effects via α2-AR [29]. Previous studies shown that Dex possess potent immune-modulatory and anti-inflammatory properties [30–32]. For instance, Dex may regulate lipopolysaccharide (LPS)-induced inflammation in murine macrophage [30]. Dex also reduced endotoxin-induced proinflammatory responses in brain microglia [31]. In addition, Dex protects septic acute kidney injury through reducing TNF-α and MCP-1 expression and inhibiting HDAC [33]. However, the effects of Dex on facilitating recovery from LPS-induced neuroinflammation and sickness behavior is unclear.

To address the role of Dex in LPS-induced neuroinflammation and sickness behavior, we examined the effect of Dex on LPS-induced microglia activation *in vivo* and *in vitro*. We also assessed the Dex affect in LPS-induced sickness behaviors and underlying mechanism.

## Materials and methods

### Animals

BALB/c mice strain adult and juvenile mice (male, 8 weeks old, 30–35 g) were purchased from BioLasco (Charles River Laboratories). All mice were housed individually in polypropylene cages and maintained in a temperature-controlled room (22 ± 2°C) on a 12 h light/dark cycle with ad libitum access to rodent chow and water except during the behavior-observation tests. At the end of each study, the mice were examined postmortem for signs of diseases such as splenomegaly or tumors. Data from mice determined to be unhealthy were excluded from analysis. All procedures were in accordance with the Taiwan National Institute of Health Guidelines for the Care and Use of Laboratory Animals and the Chi Mei Foundation Medical Center Animal Use Policy. Chi-Mei Medical Center approved the animal care protocol (Institutional Animal Care and Use Committee approval no.103072132) for the experiments in this study.

### Cell culture

BV-2 microglia cell lines purchased from the American Type Culture Collection (Manassas, USA) were cultured in RPMI 1640 supplemented with 10% fetal bovine serum (FBS) and antibiotics. The cells were maintained at 37°C in a humidified atmosphere and 5% CO_2_, and the growth medium was refreshed every two days until confluence. Cultures were washed twice and supplemented with medium containing experimental conditions.

### Behavior tests

Locomotor activity and social exploratory behavior were measured as previously described [7]. In brief, the mice were handled 2 min each day for 5days before experimentation to adapt them to routine handling. Tests were done during the dark phase (between 0800 and 1700) of the photoperiod under infrared lighting to aid video recording.

### Locomotor activity

The mice were maintained in their home cage, and locomotor activity was video-recorded during 3-min tests. On the video recordings, cages were divided into six identical rectangles and a trained observer who was blinded to experimental treatments determined the incidence of line crossing within 3 min.

### Sucrose preference

This test was done as previously described [3]. In brief, mice were provided two solutions, water or freshly prepared 2% sucrose. Fluid consumption was measured by weighing bottles before and after each test session. On the day of the sucrose preference test, mice were deprived of fluid and food for 2 h before the test [8]. At the start of the dark phase of the photoperiod, drinking water and the 2% sucrose solution were placed in the home cage for 9 h, 15 h and 21 h, respectively. At the end of each testing period, the fluid content of the conical tubes was measured and sucrose preference was determined using the equation:

Sucroseintake/Totalfluidintake(water+sucroseintake)×100%[2].

### RNA extraction and reverse transcriptase-polymerase chain reaction (RT-PCR)

Total RNA from cultured BV-2 cells and from cortex and hippocampus of mouse brain was extracted using Trizol reagent (Invitrogen, Carlsbad, CA, USA) as previously described [9]. Total mRNA (2 μg) was synthesized to cDNA using reverse transcriptase kits (Clontech, BD Biosciences, Palo Alto, CA, USA) according to the manufacturer’s protocol. RT-PCR was done on a thermal cycler (Applied Biosystems, Foster City, CA, USA) using 2x Taq DNA Polymerase Mastermix (Bioman Scientific Co., Jonghe City, New Taipei City, Taiwan). The sequences of primers used in this experiment were:

mouse TNF-α

                                forward: 5’-GAG TGA CAA GCC TGT AGC CCA-3’;
                                reverse: 5’-CCC TTC TCC AGC TGG AAG A-3’;

mouse MCP-1:

                forward: 5’-AGG TCC CTG TCA TGC TTC TG-3’;
                reverse: 5’-GCT GCT GGT GAT CCT CTT GT-3’;

mouse IDO1:

                forward: 5’-GAA GGA TCC TTG AAG ACC AC-3’;
                reverse: 5’-GAA GCT GCG ATT TCC ACC AA-3’;

mouse Bcl-2;

                forward: 5’-AGC AAC CCA ATG CCC GCT GT-3’;
                reverse: 5’-TGT GGC CCA GGT ATG CAC CCA-3’;

mouse iNOS:

                forward: 5’-TCA CCT TCG AGG GCA GCC GA-3’;
                reverse: 5’-TCC GTG GCA AAG CGA GCC AG-3’;

mouse caspase 3:

                forward: 5’-GCG GGG AGC TTG GAA CGC TA-3’;
                reverse: 5’- ACC CCG GCA GGC CTG AAT GA-3’;

mouse β-actin transcript (internal control):

                forward: 5’- GGG AAT GGG TCA GAA GGA CT-3’;
                reverse: 5’-TTT GAT GTC ACG CAC GAT TT-3’.

β-actin was used to normalize all test genes. The data were analyzed using ImageJ software (version 1.41) (National Institutes of Health, Bethesda, MD) (http://rsbweb.nih.gov/ij/), and results are expressed as fold differences.

### Real-time reverse transcriptase quantitative polymerase chain reaction (RT-qPCR)

Total RNA from cultured BV-2 cells and from cortex and hippocampus of mouse brain was extracted using Trizol reagent (Life Technologies, Rockville, MD, USA). Total RNA was subjected to reverse transcription using Strata Script H-reverse transcriptase to generate cDNA (Stratagene, La Jolla, CA, USA). To amplify the TNF-α, MCP-1, IDO1, Bcl-2, iNOS and caspase-3 transcripts, real-time PCR was done using a kit (Light Cycler-Fast Start DNA Master SYBR Green I kit; Roche Diagnostics, Indianapolis, IN, USA) according to the manufacturer’s instructions. The gene-specific primer pairs were used in real-time qPCR. Individual PCR products were analyzed using melting-point analysis. Real-time qPCR product was analyzed using the comparative Ct method according to the manufacturer’s instructions. Sample variation was corrected by subtracting the internal control gene, β-actin, Ct values from the Ct values (= ^-ΔΔ^Ct) of the genes.

### Immunohistochemical determination of Iba-1 and cleaved caspase-3

The cortex and hippocampus of each mouse brain fixed with 3.7% formaldehyde. All specimens were embedded in paraffin and sliced into 4-μm thick sections. Sections were deparaffinized and then rehydrated, antigens were retrieved, and endogenous peroxidase activity was quenched using 3% hydrogen peroxide in PBS. After the sections had been blocked with an IHC blocking reagent (Background Sniper; Biocare Medical, Concord, CA, USA) for 1 h, they were incubated with anti-Iba1 rabbit anti-mouse antibody (1:800 dilution, Biocare Medical, LLC, USA) or anti-cleaved caspase 3 rabbit anti-mouse antibody (1:500 dilution) (Cell Signaling Technology, Inc., Beverly, MA) in blocking reagent at 4°C overnight. Slides were then washed in PBS, incubated with species-specific biotinylated secondary antibody (1:200) for 30 min, washed with PBS again, amplified consecutively with avidin-horseradish peroxidase (HRP) (Vector Laboratories, Burlingame, CA, USA) and visualized by incubating them with 3,3′-diaminobenzidine tetrahydrochloride (Sigma-Aldrich, St. Louis, MO, USA). All slides were counterstained with hematoxylin (Mayer’s; ThermoShandon, Pittsburgh, PA, USA), dehydrated, and mounted. For negative controls, the procedure omitted the primary antibody.

### Western blotting

Harvested cells were lysed with a buffer containing 1% Triton X-100, 50 mM of Tris (pH 7.5), 10 mM of ethylenediamine tetraacetic acid (EDTA), 0.02% sodium azide, and a protease inhibitor cocktail (Roche Boehringer Mannheim Diagnostics, Mannheim, Germany). After one freeze-thaw cycle, cell lysates were centrifuged at 13,000 rpm for 20 min at 4°C. The lysates were boiled in sample buffer for 5 min. Protein samples (30 μg/lane) were loaded on SDS-PAGE (sodium dodecyl sulfate polyacrylamide gel electrophoresis) and electrotransferred to a polyvinylidene fluoride (PVDF) membrane (Millipore, Billerica, MA, USA). Nonspecific bindings were blocked by incubating the membrane with 5% skim milk in Tris-buffered saline (TBS) containing 0.1% Tween 20 (TBST) for 2 h. The membranes were then hybridized with primary antibodies: inducible NO synthase (iNOS, 1:500, Thermo Scientific), caspase-3 (1:200, pro-form and cleaved form, Santa Cruz, CA, USA), Bcl-xL (1:2000, Cell Signaling Technology, Inc., Beverly, MA), Iba-1 (1:1000, Biocare Medical, LLC, USA) and β-actin (1:20000, Sigma-Aldrich, Inc., USA) at 4°C overnight. The membranes were then washed with 0.1% TBST and incubated with a 1:5000 dilution of species-specific HRP-conjugated secondary antibodies (Santa Cruz Biotechnology, Santa Cruz, CA, USA) at room temperature for 1 h. After they had been washed, the membranes were soaked in electrochemiluminescence (ECL) solution (PerkinElmer Life Sciences, Boston, MA, USA) for 1 min, and then exposed to X-ray film (BioMax; Eastman Kodak, Rochester, NY, USA). The relative signal intensity was also quantified using ImageJ 1.41.

### Experimental protocols

For in vitro studies, dexmedetomidine, yohimbine hydrochloride (Sigma-Aldrich, Inc., USA) (a selective α_2_-receptor antagonist) and Escherichia coli LPS (serotype O55:B5, Sigma, St. Louis, MO) were prepared in isotonic PBS. The dose of dexmedetomidine and yohimbine used in our in vitro and in vivo experiments are based on previous reports [33, 34]. BV-2 cells were washed and replenished with medium containing 0, 1, or 10 μM dexmedetomidine. Yohimbine hydrochloride 50 μM was pre-treated combined with dexmedetomidine as indicated before LPS treatment. After 30 min, LPS 10 ng/mL was added to the culture medium for 4 hr. Total protein was collected from cell homogenates and determined using a kit (DC Protein Assay; Bio-Rad Laboratories, Hercules, CA, USA). Total RNA was isolated using Trizol reagent and the TNF-α, MCP-1, IDO1, Bcl-2, iNOS or caspase 3 were assayed using RT-PCR.

For in vivo studies, dexmedetomidine was dissolved in pyrogen-free isotonic, sterile saline. In the first study, adult male BALB/c mice were injected (i.p.) with vehicle or dexmedetomidine (50 ug/kg). yohimbine hydrochloride (250 ug/kg), the α2-adrenoceptor antagonist, was i.p. given combined with dexmedetonidine as indicated. After 30 min of dexmedetomidine with or without yohimbine administration, they were injected (i.p.) with saline or LPS (0.33 mg/kg), The dose of LPS was chosen because it caused a proinflammatory cytokine response in the brain and resulted in mild temporary sickness behavior in adult mice [35]. Twenty-four hours after LPS administration, the mice were briefly anesthetized with isoflurane and then killed using cervical dislocation. After sacrificed, their brains were removed and dissected. The cortex was stored at −80°C and the hippocampus was fixed with 3.7% formaldehyde. Total RNA was isolated from these regions using Trizol. TNF-α, MCP-1, IDO1, Bcl-2, iNOS or caspase 3 were assayed using RT-PCR.

In the second study, adult male BALB/c mice were treated with dexmedetomidine combined with or without yohimbine hydrochloride, and then LPS as described above. Their motivation to engage in social behavior was determined immediately before the injection of saline or LPS, and again 2, 4, 8, and 24 h later. Body weight, food and water intake were measured at each time point over the 24 h period. Anhedonia was evaluated using sucrose preference 24–45 h after the saline or LPS injection.

### Statistical analysis

Data were analyzed using SAS/STAT (SAS Institute, Cary, NC, USA) Generalized Linear Model (GENMOD) procedures. All data were subjected to a univariate analysis to ensure normality. Data were subjected to one, two- (Dex × LPS) or three-way (Dex × LPS × Time) ANOVA (analysis of variance) to determine significant main effects and interactions between main factors. When appropriate, a post hoc Student’s *t* test of least square means with a Tukey adjustment was used to determine whether treatment means were significantly different from one another. All statistics were performed with α = 0.05. All data are expressed as treatment means ± SEM (standard error of the mean).

## Results

### Dexmedetomidine reduced the LPS-induced sickness behavior

**Fig 1:**
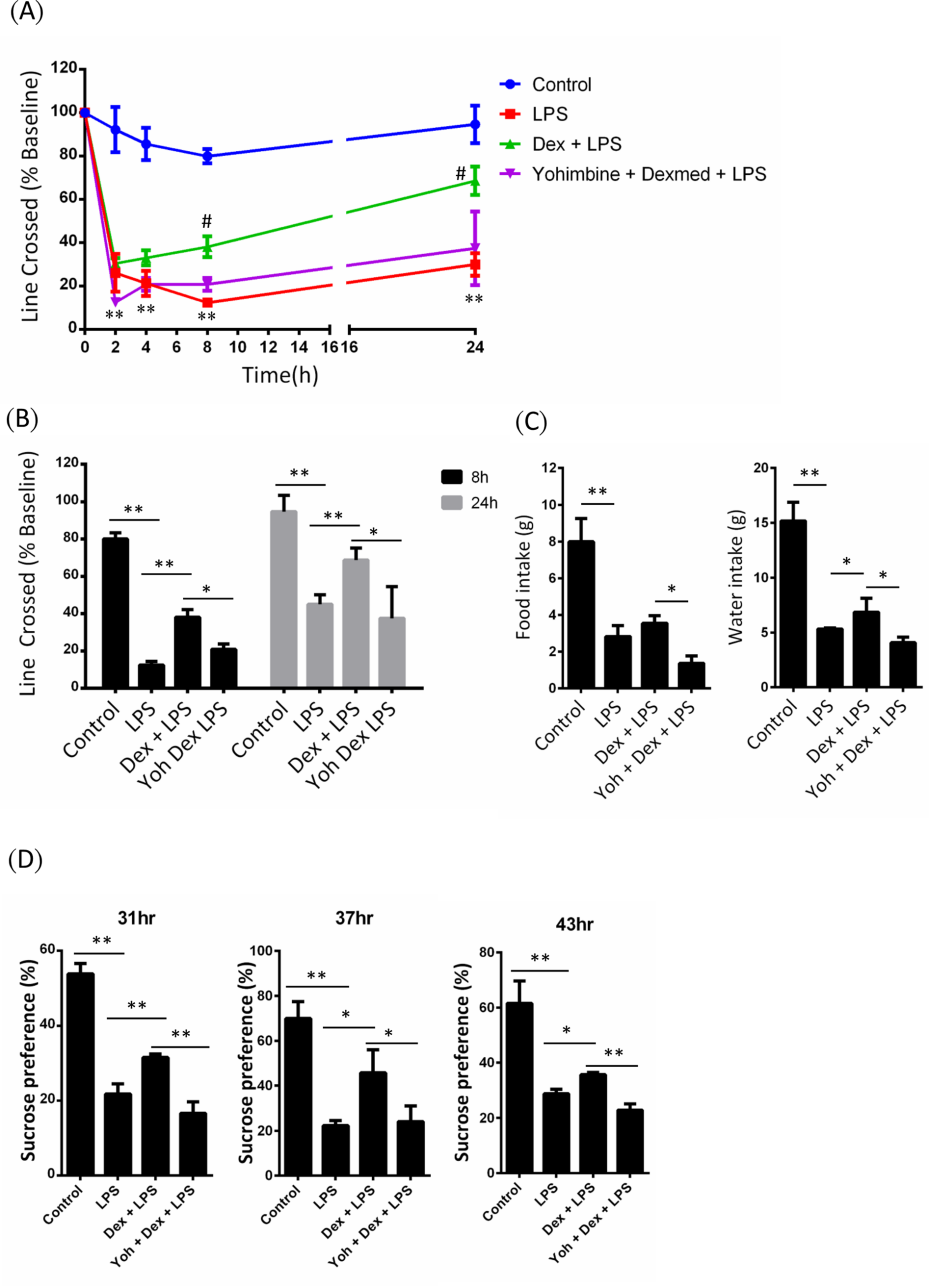
Dexmedetomidine facilitates recovery from LPS-induced sickness behavior and inhibite LPS-associated anhedonia. Mice were challenged with saline (control) or LPS or LPS combined with Dex or Dex+Yoh. (A) Social exploratory behavior was measured using line crossed percentage method before and 2, 4, 8, and 24 h after LPS injection. Data are mean ± SEM. All group n = 6. ** P < 0.005, compared with control group; # P < 0.05 compared with LPS group. (B) Line crossed percentage were measured in different groups of mice at 8 and 24 h after LPS injection. (C) Food intake and water intake were measured in 24 h after LPS injection. (D) The sucrose preference was determined at 31, 37, and 43 h after LPS injection. Data are mean ± SEM, n = 6. * P < 0.05; ** P < 0.005.

We investigated the effect of Dex on sickness response and social exploratory behavior in LPS-injected mice. We determined mice social exploratory behavior using percentage of line crossed as described in methods. After 2 hours of LPS injection, all groups of mice showed a significant reducing in line crossed (Fig 1A). At 8 and 24 h after LPS injection, LPS+Dex group showed higher line crossed percentage than LPS group (Fig 1B). The effect of Dex on LPS-induced social exploratory behavior reducing was attenuated by yohimbine. The food and water intake were reduced by LPS challenge. Dex increased water but not food intake in first 24h after LPS injection (Fig 1C). The effect of Dex on LPS-induced anhedonia was determined using sucrose preference test. The sucrose preference was observed at 31, 37 and 43 hours after LPS injection and showed that LPS significantly reduced mice sucrose preference (Fig 1D). In Dex+LPS group, sucrose preference is higher than LPS group. However, yohimbine attenuated the effects of Dex (Fig 1D).

### Dexmedetomidine reduced microglia activation in mice brain

**Fig 2:**
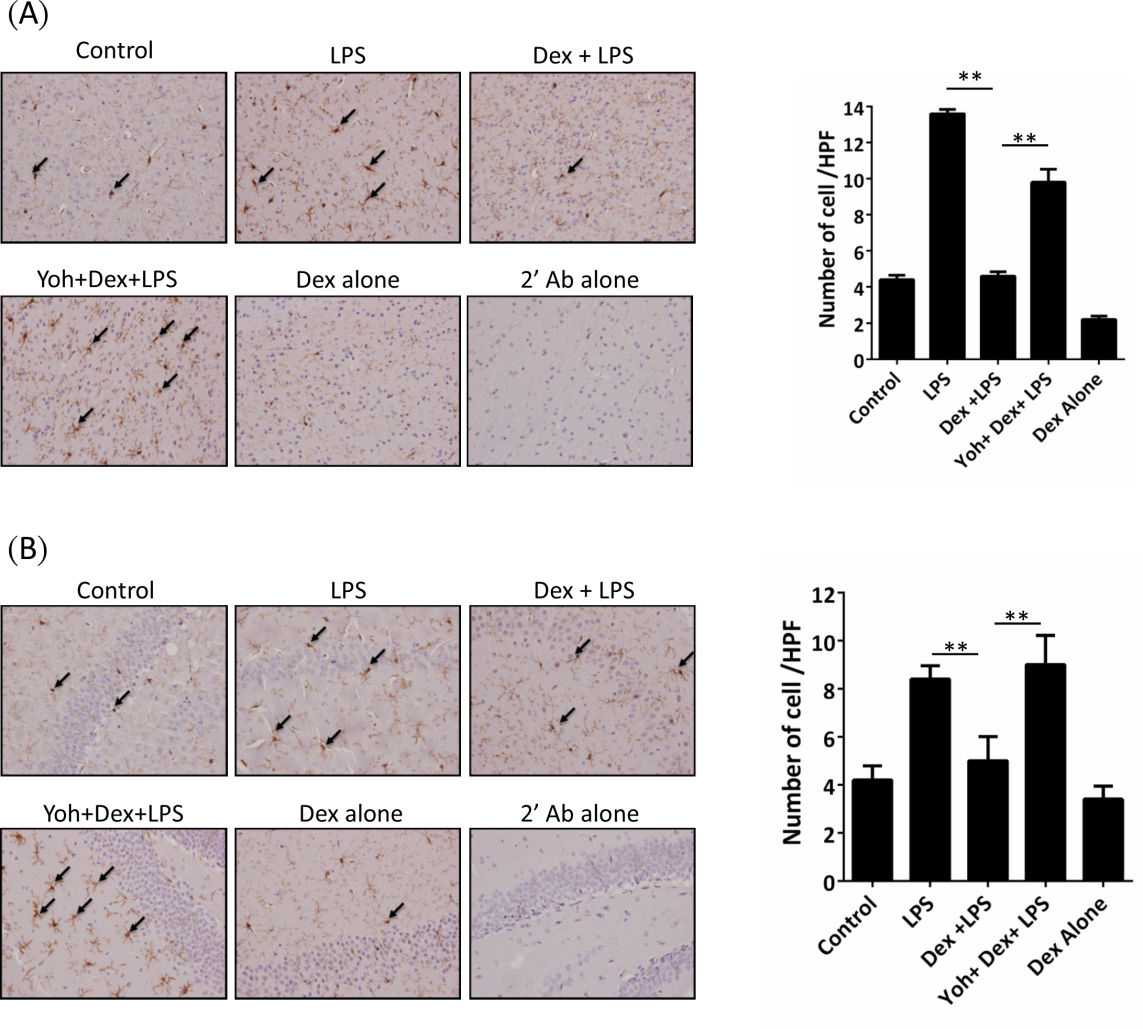
Dexmedetomidine decreased the microglia Iba-1 in the brain of LPS-challenged mice. Mice were challenged with saline (control) or LPS or LPS combined with Dex or Dex+Yoh for 24 h. Immnuohistochemistry staining showed microglia Iba-1 expression in the (A) cortex and (B) hippocampus of the mice. Dex: dexmedetomidine; Yoh: yohimbine.

**Fig 3:**
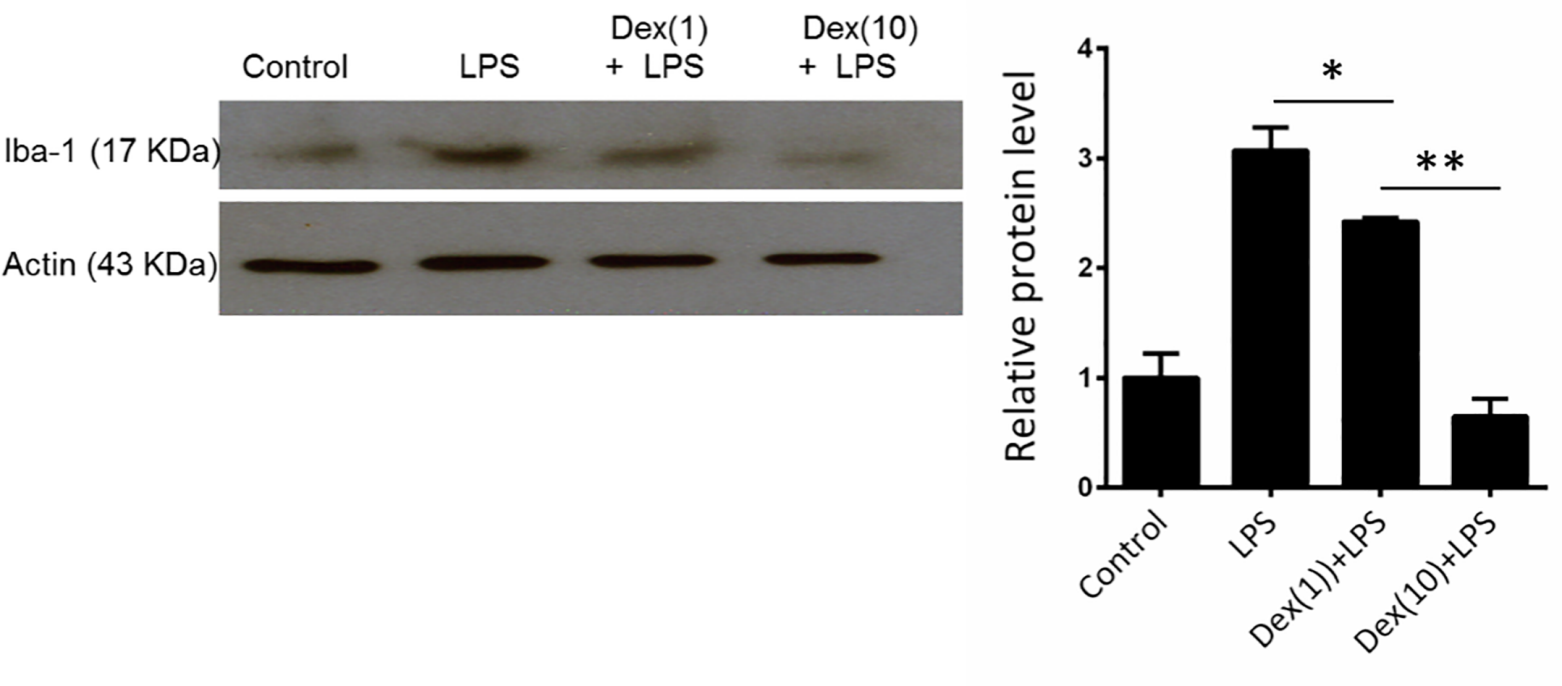
Dexmedetomidine decreased the microglia activation in BV-2 cells. BV-2 cells were treated with PBS (control) or LPS or LPS+Dex for 8 h. Iba-1 expression in the cells was determined using western blot. The right panel shows the quantification of Iba-1 levels. Dex(1): dexmedetomidine 1 uM; Dex(10): dexmedetomidine 10 uM. Data are mean ± SEM, n = 3. * P < 0.05; ** P < 0.005.

We first investigated the effect of Dex on LPS-induced mice brain microglia activation. Mice were injected with LPS combined with or without Dex pretreatment as described in methods. We determined Iba1 expression as microglia activation using IHC staining in cortex and hippocampus. In LPS-challenged mice, microglia Iba1 expression was increased in both cortex (Fig 2A) and hippocampus (Fig 2B). In mice with Dex pretreatment, LPS-induced Iba1 expression were reduced in both cortex and hippocampus regions. However, the effects of Dex on Iba1 expression were attenuated by yohimbine (Fig 2A and 2B). We next determined LPS-induced Iba1 protein levels changes in microglia cells *in vitro*. BV-2 microglia-derived cells were treated with saline or Dex and then stimulated with LPS for 8 hours. Iba1 protein levels were increased in LPS-stimulated BV-2 cells, which increasing was significantly reduced in LPS+Dex groups (Fig 3).

### Dexmedetomidine ameliorates cytokines and apoptotic-genes expression in LPS-induced neuroinflammation

**Fig 4:**
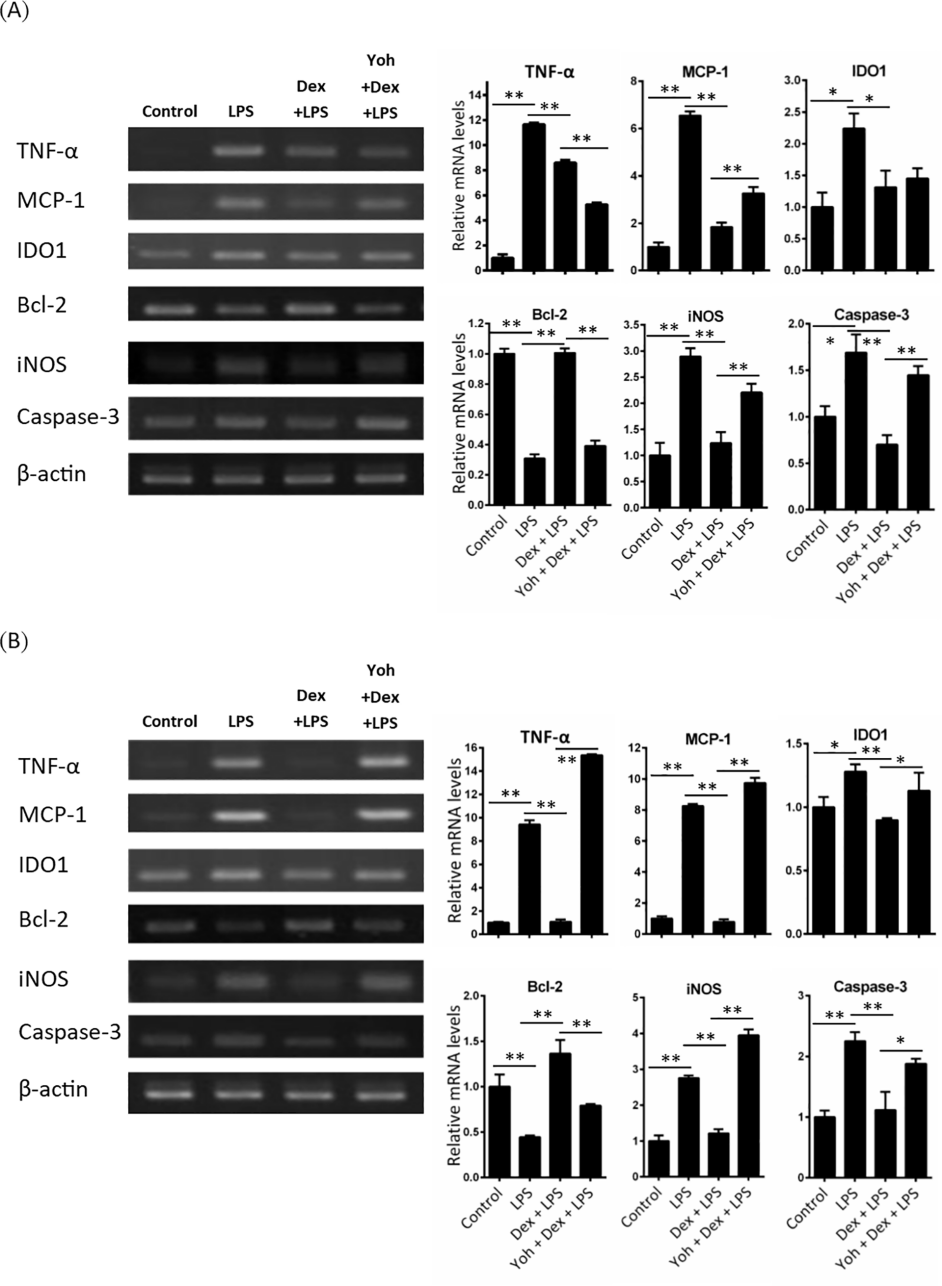
Dexmedetomidine attenuates LPS-induced mRNA expressions of inflammatory cytokines, Caspase 3, iNOs and increased Bcl-2 expression in mice brain. Mice were challenged with saline (control) or LPS or LPS combined with Dex or Dex+Yoh for 24 h. TNF-α, MCP-1, IDO1, iNOS, Bcl-2, and caspase-3 mRNA expression in (A) cortex and (B) hippocampus were determined using RT-PCR (left panel) and quantitative real-time PCR (right panel). Data are mean ± SEM, n = 6. * P < 0.05; ** P < 0.005.

Inflammatory cytokines plays an important role in neuroinflammation and in response for behavioral symptoms of sickness (e.g., anorexia, social withdrawal and anhedonia) [6]. We then investigated the effects of Dex on LPS-induced cytokines TNF-α, MCP-1, IDO1, and iNOS mRNA. Apoptotic related signals Bcl-2 and caspase 3 mRNA expression were also determined. Mice were challenged with saline or LPS with or without Dex pretreatment. After 24 h of LPS challenge, the mice were sacrificed and the TNF-α, MCP-1 and IDO1 mRNA expression in cortex and hippocampuswere were determined using RT-PCR and quantitative PCR. TNF-α, MCP-1, IDO, iNOS, and caspase 3 mRNA expression were increased but anti-apoptotic signal Bcl-2 decreased in the cortex (Fig 4A) and hippocampus (Fig 4B) of LPS-challenged mice. In mice pretreated with Dex, the LPS-induced cytokines mRNA expression were reduced in both brain regions. Yohimbine attenuated the effects of Dex on cytokines, iNOS, Bcl-2, and caspase 3 expression, which indicated the effect of Dex is specific.

**Fig 5:**
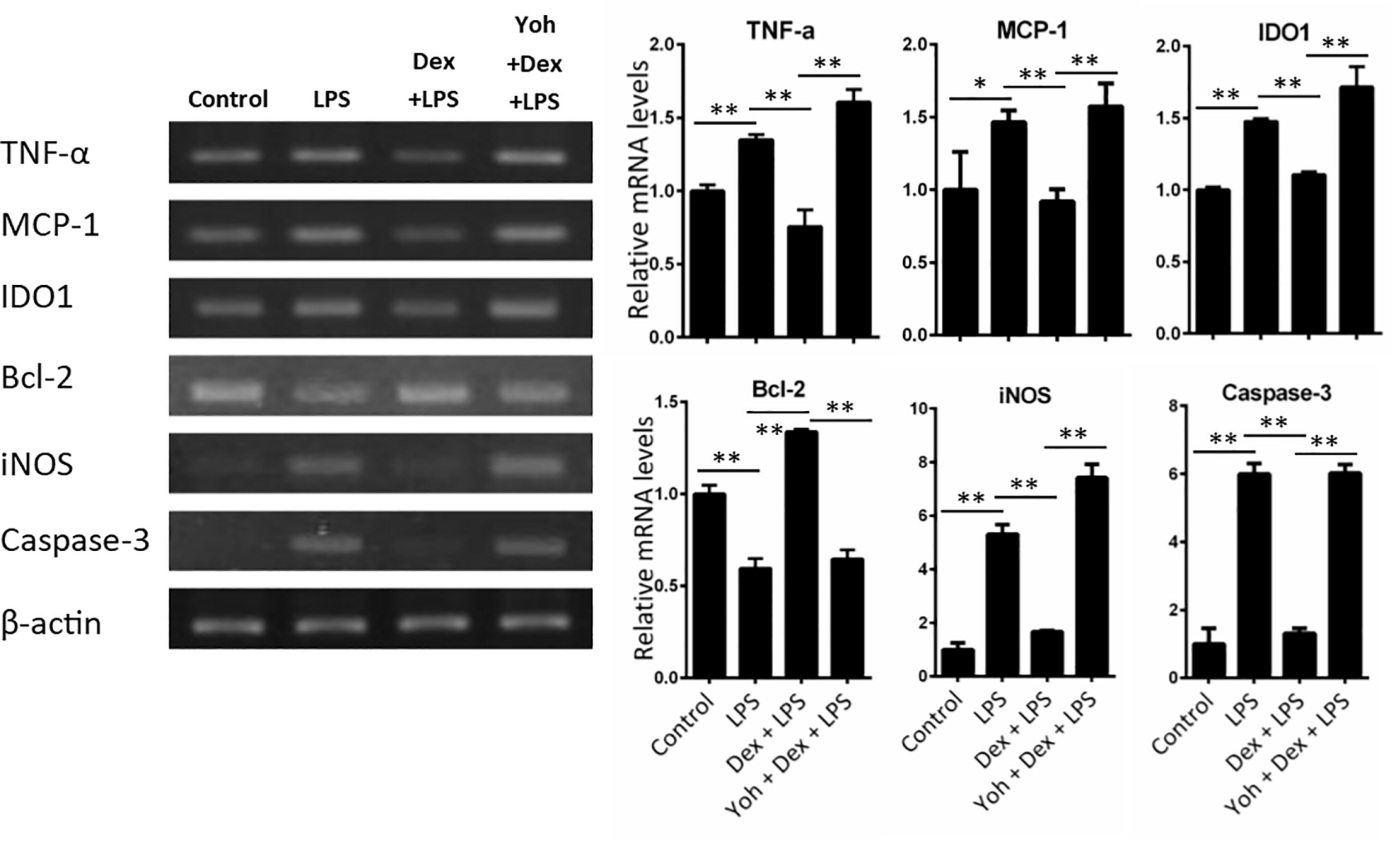
Dexmedetomidine suppresses LPS-induced mRNA expressions of inflammation cytokines, Caspase 3, iNOS and increased Bcl-2 expression in BV-2 cells. BV2 cells were challenged with PBS (control) or LPS or LPS combined with Dex or Dex+Yoh for 8 h. TNF-α, MCP-1, IDO1, Bcl-2, iNOS and caspase-3 mRNA expression were determined using RT-PCR (left panel) and quantitative real-time PCR (right panel). Data are mean ± SEM. All groups n = 3. * P < 0.05; ** P < 0.005.

In vitro, we investigated the effect of Dex in LPS-stimulated BV-2 microglia cells. BV-2 cells were stimulated with LPS with or without Dex or Dex+yohimbine pretreatment. After 6 h of LPS stimulated, the mRNA of TNF-α, MCP-1, IDO1, iNOS and Bcl2 and caspase 3 expression were determined using RT-PCR and quantitative PCR. TNF-α, MCP-1, IDO1, iNOS, and caspase 3 mRNA expression were increased but Bcl-2 decreased in LPS-stimulated BV-2 cells (Fig 5). Dex decreased LPS-induced TNF-α, MCP-1, IDO1, iNOS, and caspase 3 mRNA levels but increased Bcl-2 expression. Yohimbine ameliorated these effects of Dex in LPS-stimulated BV-2 cells.

### Dexmedetomidine reduced apoptosis in LPS-induced neuroinflammation

**Fig 6:**
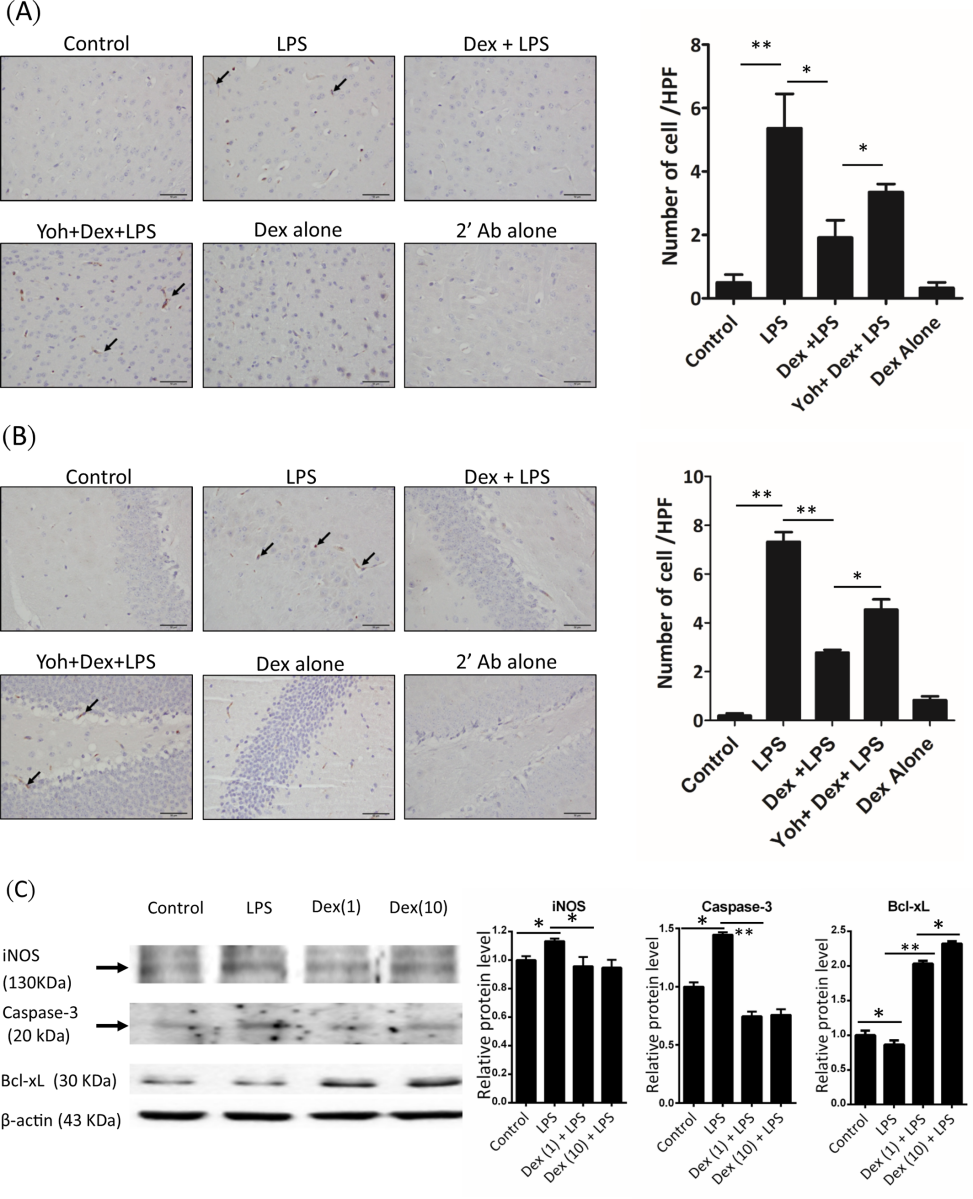
Dexmedetomidine reduced apoptotic associated signals in the LPS-challenged mice brain and BV-2 cells. Mice were challenged with saline (control) or LPS or LPS combined with Dex or Dex+Yoh for 24 h. Immnuohistochemistry staining showed cleaved caspase-3 expression (arrows) in the (A) cortex and (B) hippocampus of the mice. (C) BV-2 cells were treated with PBS (control) or LPS or LPS+Dex for 8 h. Expression of iNOS, Bcl-xL, and cleaved caspase-3 in the cells were analyzed using western blot. Dex(1): dexmedetomidine 1 uM; Dex(10): dexmedetomidine 10 uM. Data are mean ± SEM. All groups n = 3. * P < 0.05; ** P < 0.005.

We next determined cell apoptosis in LPS-challenged mice brain. Histological examination showed the cleaved caspase-3 stained cells was increased in the hippocampus and cortex of LPS-challenged mice. In Dex-pretreated mice, LPS-induced cleaved caspase-3 was reduced in both brain regions. Yohimbine ameliorates the effect of Dex on cleaved caspase-3 expression (Fig 6A and 6B). In vitro, we determined the effects of Dex on LPS-stimulated BV-2 cells in protein levels of apoptotic signals. BV-2 cells were stimulated with LPS combined with or without Dex for 8 h. LPS increased iNOS expression which was reduced in LPS+Dex groups. The cleaved caspase-3 was increased in LPS group but decreased in LPS+Dex groups. The anti-apoptotic protein Bcl-xL was increased in LPS+Dex groups (Fig 6C).

## Discussion

Neuroinflammation is associated with a myriad of complications including prolonged sickness and depressive-like behavior [13, 36]. Here we showed that Dex was effective in facilitating the recovery from LPS-induced neuroinflammation and sickness behavior. Dex treatment inhibited the LPS-induced microglia activation. The LPS-induced inflammatory cytokines, TNF-α, MCP-1 and IDO1 expression was reduced by Dex treatment. Dex also affected apoptosis associated proteins, Bcl-2, Bcl-xL and caspase-3 in LPS inflammation.

Dex is a specific α2-adrenoceptor (α2-AR) agonist and has been showed with anti-inflammatory effects [37, 38]. Paminoclonidine and clonidine, another two α2-AR agonists, suppressed IL-6 and TNF production in in vitro study [37, 38]. Recent studies showed that Dex decreased inflammation and increased survival rate in sepsis and endotoxin-induced shock animal model [39]. Our previous study also showed Dex protects septic acute kidney injury through reduced cytokines TNF-α and MCP-1 production [33]. Dex also reduces cytokines production IL-1β, TNF-α, and IL-6 in microglia cell [31]. In animal study, the expression of TNF-α, MCP-1 in brain was induced through systemic administration of LPS, which also affected sickness behavior [36]. In this study we showed Dex reduced cytokines production and improved sickness behavior in LPS- treated mice.

The α2 receptors constitute a family of G-protein–coupled receptors (GPCR) with three pharmacological subtypes, α-2A, α-2B, and α-2C. The α-2A and -2C subtypes are found mainly in the central nervous system. Stimulation of these receptor subtypes are responsible for sedation, analgesia and sympatholytic effects [40]. The α-2B receptors are found more frequently on vascular smooth muscle and have been shown to mediate vasopressor effects. All 3 subtypes inhibit adenylyl cyclase, reducing the levels of cyclic adenosine monophosphate (cAMP) and causing hyperpolarization of noradrenergic neurons. As cAMP is inhibited, potassium efflux through calcium-activated channels in turn prevents calcium ions from entering the nerve terminal, thus, leading to a suppression of neural firing. This suppression inhibits norepinephrine release and reduces activity of the ascending noradrenergic pathways, resulting in sedation and hypnosis [41]. In addition, activated α2 receptors also inhibited neuroinflammation and reduced cell apoptosis in cortex and hippocampus as shown in our results. However, whether the anti-inflammation and anti-apoptosis effects were mediated through GPCR/cAMP/ion channels activity needs to be further investigated.

Central nervous system can be affected by peripheral immune responses [42]. Inflammatory cytokines induced by peripheral LPS treatment may be forwarded to the brain and activate microglia and associated with sickness behavior [42]. The activation of microglia in the brain of septic patients also play an important role in behavioral changes associated with systemic infection [43]. Iba1 is specifically expressed and plays a role in the activated microglia. Iba1 is a microglia-activation marker in LPS-mediated inflammatory response [44, 45]. Previous studies suggested the anti-inflammatory effects of Dex on systemic inflammation [30, 39]. Here, our data showed the Dex suppress Iba-1 expression in LPS-induced mice and BV2 cells. We suggested that Dex improves cytokine-induced sickness behavior through regulate systemic inflammation and neuroinflammation.

IDO is significantly increased in response to inflammation and involved in neuroinflammatory disease[46]. Modulate the IDO activation can reduced LPS-induced depressive like behavior or anhedonia [46]. Here we show that IDO mRNA expression is ameliorated by Dex in LPS-treated mice and BV2 cell, which supports the role of IDO in LPS-induced anhedonia and partly explains the effect of Dex on LPS neuroinflammation.

The inflammatory mediators such as TNF-α, MCP-1 and iNOS derived from microglia cells are involving in the neurodegeneration and neuronal cell death [20, 25]. Oxidative stress is implicated in neuroinflammation. The NO from iNOS induces ROS in neurons and cause s cognitive impairment. The iNOS is upregulated in LPS-stimulated mcroglia cell [20, 47, 48]. In this present data iNOS was reduced in Dex-treated mice and BV2 cells. We thus considered that Dex may regulate ROS activation through inhibiting iNOS expression.

Apoptotic-associated genes Bcl-2, Bcl-xL and caspase-3 are involved in LPS-induced neuroinflammation [25]. Caspase-3 is also activated in neurological disorders. Previous studies showed that cleaved caspase-3 is associated with apoptosis of neurons and neuroinflammation [49, 50]. The derived TNF-α from activated microglia induced neural precursor cells apoptosis via the Bcl-2 family protein Bax.

Dex suppressed the hippocampus apoptosis in intracerebral hemorrhage rat brain injury through regulated caspase-3 and Bcl-2 expression and ameliorated memory impairment [51]. In this study, we explored that dexmedetomidine is antiapoptotic. Dex reduced caspase-3 mRNA expression and increased Bcl-2 mRNA expression in vivo and in vitro. Previous studies suggested that administration of an α2-AR agonist during the critical phase of synaptogenesis activates the endogenous postsynaptic norepinephrine-mediated trophic system, which couples to a Bcl-2 antiapoptotic effect [52]. We also showed that Dex induced Bcl-xL expression in LPS-treated BV2 cells. We supposed that Dex improves LPS-induced sickness behavior via regulated neurons cell apoptosis. Taking all our results together, we found that Dex regulated apoptotic-associated gene and cytokine expression in LPS-treated mice and microglial cells. Dex improved LPS-induced neuroinflammation and sickness behavior in mice. The mechanism of Dex in regulating apoptotic-associated gene expression requires further investigation.

## Conclusion

This study showed that Dex decreases LPS-induced sickness behavior symptoms, anorexia and anhedonia, through ameliorates microglia activation and production of neuroinflammatory mediators. Dex can be used to mitigate cytokine expression in the brain and beneficially affect mood, motivation, and behavior. Dex may be a beneficial part of a pharmacological strategy to decrease infection-induced neuroinflammation and sickness behaviors.

## References

[1] Dantzer R, Kelley KW. Twenty years of research on cytokine-induced sickness behavior. Brain Behav Immun. 2007;21(2):153–60. Epub 2006/11/08. doi: 10.1016/j.bbi.2006.09.006 ; PubMed Central PMCID: PMCPmc1850954. 17088043 10.1016/j.bbi.2006.09.006 PMC1850954

[2] Skalisz LL, Beijamini V, Joca SL, Vital MA, Da Cunha C, Andreatini R. Evaluation of the face validity of reserpine administration as an animal model of depression—Parkinson's disease association. Prog Neuropsychopharmacol Biol Psychiatry. 2002;26(5):879–83. Epub 2002/10/09. . 12369260 10.1016/s0278-5846(01)00333-5

[3] Frenois F, Moreau M, O'Connor J, Lawson M, Micon C, Lestage J, et al Lipopolysaccharide induces delayed FosB/DeltaFosB immunostaining within the mouse extended amygdala, hippocampus and hypothalamus, that parallel the expression of depressive-like behavior. Psychoneuroendocrinology. 2007;32(5):516–31. Epub 2007/05/08. doi: 10.1016/j.psyneuen.2007.03.005 ; PubMed Central PMCID: PMCPmc1978247. 17482371 10.1016/j.psyneuen.2007.03.005 PMC1978247

[4] Schrier RW, Wang W. Acute renal failure and sepsis. N Engl J Med. 2004;351(2):159–69. Epub 2004/07/13. doi: 10.1056/NEJMra032401 . 15247356 10.1056/NEJMra032401

[5] Iwashyna TJ, Ely EW, Smith DM, Langa KM. Long-term cognitive impairment and functional disability among survivors of severe sepsis. Jama. 2010;304(16):1787–94. Epub 2010/10/28. doi: 10.1001/jama.2010.1553 ; PubMed Central PMCID: PMCPmc3345288. 20978258 10.1001/jama.2010.1553 PMC3345288

[6] Kelley KW, Bluthe RM, Dantzer R, Zhou JH, Shen WH, Johnson RW, et al Cytokine-induced sickness behavior. Brain Behav Immun. 2003;17 Suppl 1:S112–8. Epub 2003/03/05. . 12615196 10.1016/s0889-1591(02)00077-6

[7] Abraham J, Jang S, Godbout JP, Chen J, Kelley KW, Dantzer R, et al Aging sensitizes mice to behavioral deficits induced by central HIV-1 gp120. Neurobiol Aging. 2008;29(4):614–21. Epub 2006/12/19. doi: 10.1016/j.neurobiolaging.2006.11.002 ; PubMed Central PMCID: PMCPmc2374923. 17174449 10.1016/j.neurobiolaging.2006.11.002 PMC2374923

[8] Craft TK, DeVries AC. Role of IL-1 in poststroke depressive-like behavior in mice. Biol Psychiatry. 2006;60(8):812–8. Epub 2006/05/30. doi: 10.1016/j.biopsych.2006.03.011 . 16730336 10.1016/j.biopsych.2006.03.011

[9] Laye S, Parnet P, Goujon E, Dantzer R. Peripheral administration of lipopolysaccharide induces the expression of cytokine transcripts in the brain and pituitary of mice. Brain Res Mol Brain Res. 1994;27(1):157–62. Epub 1994/11/01. . 7877446 10.1016/0169-328x(94)90197-x

[10] Dantzer R, O'Connor JC, Freund GG, Johnson RW, Kelley KW. From inflammation to sickness and depression: when the immune system subjugates the brain. Nat Rev Neurosci. 2008;9(1):46–56. Epub 2007/12/13. doi: 10.1038/nrn2297 ; PubMed Central PMCID: PMCPmc2919277. 18073775 10.1038/nrn2297 PMC2919277

[11] D'Mello C, Le T, Swain MG. Cerebral microglia recruit monocytes into the brain in response to tumor necrosis factoralpha signaling during peripheral organ inflammation. J Neurosci. 2009;29(7):2089–102. Epub 2009/02/21. doi: 10.1523/JNEUROSCI.3567-08.2009 . 19228962 10.1523/JNEUROSCI.3567-08.2009 PMC6666330

[12] Chen J, Buchanan JB, Sparkman NL, Godbout JP, Freund GG, Johnson RW. Neuroinflammation and disruption in working memory in aged mice after acute stimulation of the peripheral innate immune system. Brain Behav Immun. 2008;22(3):301–11. Epub 2007/10/24. doi: 10.1016/j.bbi.2007.08.014 ; PubMed Central PMCID: PMCPmc2374919. 17951027 10.1016/j.bbi.2007.08.014 PMC2374919

[13] Huang Y, Henry CJ, Dantzer R, Johnson RW, Godbout JP. Exaggerated sickness behavior and brain proinflammatory cytokine expression in aged mice in response to intracerebroventricular lipopolysaccharide. Neurobiol Aging. 2008;29(11):1744–53. Epub 2007/06/05. doi: 10.1016/j.neurobiolaging.2007.04.012 ; PubMed Central PMCID: PMCPmc2647751. 17543422 10.1016/j.neurobiolaging.2007.04.012 PMC2647751

[14] Godbout JP, Moreau M, Lestage J, Chen J, Sparkman NL, O'Connor J, et al Aging exacerbates depressive-like behavior in mice in response to activation of the peripheral innate immune system. Neuropsychopharmacology. 2008;33(10):2341–51. Epub 2007/12/14. doi: 10.1038/sj.npp.1301649 ; PubMed Central PMCID: PMCPmc2907915. 18075491 10.1038/sj.npp.1301649 PMC2907915

[15] Garden GA, Moller T. Microglia biology in health and disease. J Neuroimmune Pharmacol. 2006;1(2):127–37. Epub 2007/11/28. doi: 10.1007/s11481-006-9015-5 . 18040779 10.1007/s11481-006-9015-5

[16] Kraft AD, Harry GJ. Features of microglia and neuroinflammation relevant to environmental exposure and neurotoxicity. Int J Environ Res Public Health. 2011;8(7):2980–3018. Epub 2011/08/17. doi: 10.3390/ijerph8072980 ; PubMed Central PMCID: PMCPmc3155341. 21845170 10.3390/ijerph8072980 PMC3155341

[17] Saijo K, Glass CK. Microglial cell origin and phenotypes in health and disease. Nat Rev Immunol. 2011;11(11):775–87. Epub 2011/10/26. doi: 10.1038/nri3086 . 22025055 10.1038/nri3086

[18] Imai Y, Ibata I, Ito D, Ohsawa K, Kohsaka S. A novel gene iba1 in the major histocompatibility complex class III region encoding an EF hand protein expressed in a monocytic lineage. Biochem Biophys Res Commun. 1996;224(3):855–62. doi: 10.1006/bbrc.1996.1112 . 8713135 10.1006/bbrc.1996.1112

[19] Ohsawa K, Imai Y, Kanazawa H, Sasaki Y, Kohsaka S. Involvement of Iba1 in membrane ruffling and phagocytosis of macrophages/microglia. J Cell Sci. 2000;113 (Pt 17):3073–84. . 10934045 10.1242/jcs.113.17.3073

[20] Golden TR, Patel M. Catalytic antioxidants and neurodegeneration. Antioxid Redox Signal. 2009;11(3):555–70. Epub 2008/08/30. doi: 10.1089/ARS.2008.2256 ; PubMed Central PMCID: PMCPmc2933572. 18754709 10.1089/ars.2008.2256 PMC2933572

[21] Banati RB, Gehrmann J, Schubert P, Kreutzberg GW. Cytotoxicity of microglia. Glia. 1993;7(1):111–8. Epub 1993/01/01. doi: 10.1002/glia.440070117 . 8423058 10.1002/glia.440070117

[22] Cunningham C, Campion S, Lunnon K, Murray CL, Woods JF, Deacon RM, et al Systemic inflammation induces acute behavioral and cognitive changes and accelerates neurodegenerative disease. Biol Psychiatry. 2009;65(4):304–12. Epub 2008/09/20. doi: 10.1016/j.biopsych.2008.07.024 ; PubMed Central PMCID: PMCPmc2633437. 18801476 10.1016/j.biopsych.2008.07.024 PMC2633437

[23] Godbout JP, Chen J, Abraham J, Richwine AF, Berg BM, Kelley KW, et al Exaggerated neuroinflammation and sickness behavior in aged mice following activation of the peripheral innate immune system. Faseb j. 2005;19(10):1329–31. Epub 2005/05/28. doi: 10.1096/fj.05-3776fje . 15919760 10.1096/fj.05-3776fje

[24] De La Garza R 2nd. Endotoxin- or pro-inflammatory cytokine-induced sickness behavior as an animal model of depression: focus on anhedonia. Neurosci Biobehav Rev. 2005;29(4–5):761–70. Epub 2005/05/10. doi: 10.1016/j.neubiorev.2005.03.016 . 15878621 10.1016/j.neubiorev.2005.03.016

[25] Guadagno J, Xu X, Karajgikar M, Brown A, Cregan SP. Microglia-derived TNFalpha induces apoptosis in neural precursor cells via transcriptional activation of the Bcl-2 family member Puma. Cell Death Dis. 2013;4:e538 Epub 2013/03/16. doi: 10.1038/cddis.2013.59 ; PubMed Central PMCID: PMCPmc3613837. 23492769 10.1038/cddis.2013.59 PMC3613837

[26] Gertler R, Brown HC, Mitchell DH, Silvius EN. Dexmedetomidine: a novel sedative-analgesic agent. Proc (Bayl Univ Med Cent). 2001;14(1):13–21. Epub 2005/12/22. ; PubMed Central PMCID: PMCPmc1291306. 16369581 10.1080/08998280.2001.11927725 PMC1291306

[27] Aantaa R, Jalonen J. Perioperative use of alpha2-adrenoceptor agonists and the cardiac patient. Eur J Anaesthesiol. 2006;23(5):361–72. Epub 2006/03/02. doi: 10.1017/S0265021506000378 . 16507202 10.1017/S0265021506000378

[28] Shukry M, Clyde MC, Kalarickal PL, Ramadhyani U. Does dexmedetomidine prevent emergence delirium in children after sevoflurane-based general anesthesia? Paediatr Anaesth. 2005;15(12):1098–104. doi: 10.1111/j.1460-9592.2005.01660.x . 16324031 10.1111/j.1460-9592.2005.01660.x

[29] Ma D, Hossain M, Rajakumaraswamy N, Arshad M, Sanders RD, Franks NP, et al Dexmedetomidine produces its neuroprotective effect via the alpha 2A-adrenoceptor subtype. European journal of pharmacology. 2004;502(1–2):87–97. Epub 2004/10/07. doi: 10.1016/j.ejphar.2004.08.044 . 15464093 10.1016/j.ejphar.2004.08.044

[30] Lai YC, Tsai PS, Huang CJ. Effects of dexmedetomidine on regulating endotoxin-induced up-regulation of inflammatory molecules in murine macrophages. J Surg Res. 2009;154(2):212–9. Epub 2009/02/03. doi: 10.1016/j.jss.2008.07.010 . 19181340 10.1016/j.jss.2008.07.010

[31] Peng M, Wang YL, Wang CY, Chen C. Dexmedetomidine attenuates lipopolysaccharide-induced proinflammatory response in primary microglia. J Surg Res. 2013;179(1):e219–25. Epub 2012/06/12. doi: 10.1016/j.jss.2012.05.047 . 22683080 10.1016/j.jss.2012.05.047

[32] Xiang H, Hu B, Li Z, Li J. Dexmedetomidine controls systemic cytokine levels through the cholinergic anti-inflammatory pathway. Inflammation. 2014;37(5):1763–70. Epub 2014/05/08. doi: 10.1007/s10753-014-9906-1 . 24803295 10.1007/s10753-014-9906-1

[33] Hsing CH, Lin CF, So E, Sun DP, Chen TC, Li CF, et al alpha2-Adrenoceptor agonist dexmedetomidine protects septic acute kidney injury through increasing BMP-7 and inhibiting HDAC2 and HDAC5. Am J Physiol Renal Physiol. 2012;303(10):F1443–53. doi: 10.1152/ajprenal.00143.2012 . 22933299 10.1152/ajprenal.00143.2012

[34] Sanders RD, Xu J, Shu Y, Januszewski A, Halder S, Fidalgo A, et al Dexmedetomidine attenuates isoflurane-induced neurocognitive impairment in neonatal rats. Anesthesiology. 2009;110(5):1077–85. doi: 10.1097/ALN.0b013e31819daedd . 19352168 10.1097/ALN.0b013e31819daedd

[35] Bret-Dibat JL, Dantzer R. Cholecystokinin receptors do not mediate the suppression of food-motivated behavior by lipopolysaccharide and interleukin-1 beta in mice. Physiol Behav. 2000;69(3):325–31. Epub 2000/06/28. . 10869599 10.1016/s0031-9384(00)00212-2

[36] Biesmans S, Meert TF, Bouwknecht JA, Acton PD, Davoodi N, De Haes P, et al Systemic immune activation leads to neuroinflammation and sickness behavior in mice. Mediators of inflammation. 2013;2013:271359 Epub 2013/08/13. doi: 10.1155/2013/271359 ; PubMed Central PMCID: PMCPmc3723093. 23935246 10.1155/2013/271359 PMC3723093

[37] Straub RH, Herrmann M, Berkmiller G, Frauenholz T, Lang B, Scholmerich J, et al Neuronal regulation of interleukin 6 secretion in murine spleen: adrenergic and opioidergic control. J Neurochem. 1997;68(4):1633–9. Epub 1997/04/01. . 9084435 10.1046/j.1471-4159.1997.68041633.x

[38] Maes M, Lin A, Kenis G, Egyed B, Bosmans E. The effects of noradrenaline and alpha-2 adrenoceptor agents on the production of monocytic products. Psychiatry Res. 2000;96(3):245–53. Epub 2000/11/21. . 11084220 10.1016/s0165-1781(00)00216-x

[39] Taniguchi T, Kidani Y, Kanakura H, Takemoto Y, Yamamoto K. Effects of dexmedetomidine on mortality rate and inflammatory responses to endotoxin-induced shock in rats. Crit Care Med. 2004;32(6):1322–6. Epub 2004/06/10. . 15187514 10.1097/01.ccm.0000128579.84228.2a

[40] Buerkle H, Yaksh TL. Pharmacological evidence for different alpha 2-adrenergic receptor sites mediating analgesia and sedation in the rat. Br J Anaesth. 1998;81(2):208–15. . 9813525 10.1093/bja/81.2.208

[41] Carollo DS, Nossaman BD, Ramadhyani U. Dexmedetomidine: a review of clinical applications. Curr Opin Anaesthesiol. 2008;21(4):457–61. doi: 10.1097/ACO.0b013e328305e3ef . 18660652 10.1097/ACO.0b013e328305e3ef

[42] Perry VH. The influence of systemic inflammation on inflammation in the brain: implications for chronic neurodegenerative disease. Brain Behav Immun. 2004;18(5):407–13. Epub 2004/07/22. doi: 10.1016/j.bbi.2004.01.004 . 15265532 10.1016/j.bbi.2004.01.004

[43] Lemstra AW, Groen in't Woud JC, Hoozemans JJ, van Haastert ES, Rozemuller AJ, Eikelenboom P, et al Microglia activation in sepsis: a case-control study. J Neuroinflammation. 2007;4:4 Epub 2007/01/17. doi: 10.1186/1742-2094-4-4 ; PubMed Central PMCID: PMCPmc1783646. 17224051 10.1186/1742-2094-4-4 PMC1783646

[44] Imai Y, Kohsaka S. Intracellular signaling in M-CSF-induced microglia activation: role of Iba1. Glia. 2002;40(2):164–74. Epub 2002/10/16. doi: 10.1002/glia.10149 . 12379904 10.1002/glia.10149

[45] Ito D, Imai Y, Ohsawa K, Nakajima K, Fukuuchi Y, Kohsaka S. Microglia-specific localisation of a novel calcium binding protein, Iba1. Brain Res Mol Brain Res. 1998;57(1):1–9. Epub 1998/06/19. . 9630473 10.1016/s0169-328x(98)00040-0

[46] O'Connor JC, Lawson MA, Andre C, Moreau M, Lestage J, Castanon N, et al Lipopolysaccharide-induced depressive-like behavior is mediated by indoleamine 2,3-dioxygenase activation in mice. Mol Psychiatry. 2009;14(5):511–22. Epub 2008/01/16. doi: 10.1038/sj.mp.4002148 ; PubMed Central PMCID: PMCPmc2683474. 18195714 10.1038/sj.mp.4002148 PMC2683474

[47] Hernandes MS, D'Avila JC, Trevelin SC, Reis PA, Kinjo ER, Lopes LR, et al The role of Nox2-derived ROS in the development of cognitive impairment after sepsis. J Neuroinflammation. 2014;11:36 Epub 2014/02/28. doi: 10.1186/1742-2094-11-36 ; PubMed Central PMCID: PMCPmc3974031. 24571599 10.1186/1742-2094-11-36 PMC3974031

[48] Lei B, Mace B, Dawson HN, Warner DS, Laskowitz DT, James ML. Anti-inflammatory effects of progesterone in lipopolysaccharide-stimulated BV-2 microglia. PloS one. 2014;9(7):e103969 Epub 2014/08/01. doi: 10.1371/journal.pone.0103969 ; PubMed Central PMCID: PMCPmc4117574. 25080336 10.1371/journal.pone.0103969 PMC4117574

[49] Burguillos MA, Deierborg T, Kavanagh E, Persson A, Hajji N, Garcia-Quintanilla A, et al Caspase signalling controls microglia activation and neurotoxicity. Nature. 2011;472(7343):319–24. Epub 2011/03/11. doi: 10.1038/nature09788 . 21389984 10.1038/nature09788

[50] Venero JL, Burguillos MA, Joseph B. Caspases playing in the field of neuroinflammation: old and new players. Dev Neurosci. 2013;35(2–3):88–101. Epub 2013/03/01. doi: 10.1159/000346155 . 23445938 10.1159/000346155

[51] Hwang L, Choi IY, Kim SE, Ko IG, Shin MS, Kim CJ, et al Dexmedetomidine ameliorates intracerebral hemorrhage-induced memory impairment by inhibiting apoptosis and enhancing brain-derived neurotrophic factor expression in the rat hippocampus. Int J Mol Med. 2013;31(5):1047–56. Epub 2013/03/19. doi: 10.3892/ijmm.2013.1301 . 23503673 10.3892/ijmm.2013.1301

[52] Dahmani S, Paris A, Jannier V, Hein L, Rouelle D, Scholz J, et al Dexmedetomidine increases hippocampal phosphorylated extracellular signal-regulated protein kinase 1 and 2 content by an alpha 2-adrenoceptor-independent mechanism: evidence for the involvement of imidazoline I1 receptors. Anesthesiology. 2008;108(3):457–66. doi: 10.1097/ALN.0b013e318164ca81 . 18292683 10.1097/ALN.0b013e318164ca81

